# Prospective Study on Splinting for First Carpometacarpal Joint: A Comparison of Conventional and Three-Dimensional Printed Splint

**DOI:** 10.3390/jcm13237043

**Published:** 2024-11-22

**Authors:** Naoto Inaba, Takuji Iwamoto, Kazunori Ishii, Satoshi Oki, Taku Suzuki, Kazuki Sato, Takeo Nagura, Masaya Nakamura

**Affiliations:** 1 Department of Orthopaedic Surgery, Keio University School of Medicine, Tokyo 160-8582, Japan; 2 Department of Clinical Biomechanics, Surgery, Keio University School of Medicine, Tokyo 160-8582, Japan; 3 Institute for Integrated Sports Medicine, Keio University School of Medicine, Tokyo 160-8582, Japan

**Keywords:** carpometacarpal, computed tomography, splint, three-dimensional printing

## Abstract

**Background**: Patient compliance is a major concern of hand orthosis in first carpometacarpal osteoarthritis. To address this issue, we established a method for creating a custom-made three-dimensional printed splint based on computed tomography. This prospective study evaluates the usefulness of the three-dimensional printed splint compared with the conventional splint. **Methods**: A total of 12 hands in nine patients were included. The mean age of the patients was 69 years (range: 58–84). Conventional orthoses were made by prosthetists using molds. Three-dimensional printed orthoses (long and short types) were digitally designed from computed tomography data and created using Fused Deposition Modeling. Subjects were instructed to use three types of orthoses for 2 weeks each. They completed questionnaires that indicated pain, function, percentage of daytime spent using the orthosis, satisfaction score, and discomfort caused by wearing orthoses. **Results**: The pain on motion showed an improvement of approximately 20% for all orthoses. There was no significant difference in pain scale, function, percentages of daytime spent using each orthosis, and satisfaction score among the three types of orthoses. Discomfort caused by wearing orthosis was more frequent in conventional orthosis than in 3D-printed orthosis, and there was a significant difference between the conventional type and the long-type 3D-printed orthosis. **Conclusions**: This study suggests that 3D-printed splints provide comparable pain relief to conventional splints with reduced discomfort. However, limitations such as small sample size, short follow-up, and reliance on CT imaging highlight the need for further research.

## 1. Introduction

Osteoarthritis of the first carpometacarpal joint (first CMC-OA) is a common degenerative disease of the hand, especially in middle-aged and elderly women [1]. In a population-based cohort study of 1535 subjects with an average age of 65 years, 50% had arthritic changes in the first CMC joint on radiographs [2]. The role of the thumb in activities of daily living (ADL) is extremely crucial, and pain in the thumb has a significant impact on ADLs. Surgical treatment includes arthrodesis [3], excision of the trapezium [4], implant arthroplasty [5], first metacarpal abduction osteotomy [6], and trapeziectomy with ligament reconstruction and tendon interposition [7] are widely performed. However, non-operative treatment, including hand orthosis, remains the most important initial treatment. Hand orthosis is effective for the first CMC-OA and is the best conservative treatment for patients who can wear it for several months [1,8,9]. In a systematic review of the conservative treatment for the first CMC-OA, evidence of the effectiveness of multiple orthoses is observed, although the quality of evidence is poor compared to surgery [10]. A variety of orthoses have been employed; however, it remains unclear which type of orthoses is the best in terms of material, shape, and area to be fixed. Additionally, poor compliance is a major problem in hand orthosis [11]. Numerous patients stop using rigid orthoses in a short period because it is difficult to perform ADLs while wearing a rigid orthosis. Improvement of compliance in the initial period of use is crucial for hand orthosis.

When making a conventional custom-made orthosis, the molding process is performed manually by the prosthetist. Hence, the suitability of the orthosis depends on the skill of the prosthetist. Sometimes the fit is poor and may require repeated adjustments. Therefore, we have established a method to create a novel custom-made three-dimensional printed orthosis (3D-printed orthosis) by computer-aided design (CAD) and computer-aided manufacturing (CAM). This is based on computed tomography (CT) of the images of individual patients. We hypothesized that CT data provide accurate digital surface skin data and the position of the joints for each patient. This enables highly reproducible designs, regardless of the experience and skill of the prosthetist. Additionally, the use of 3D-printed filaments is more flexible than the plastics used in conventional orthosis and is expected to improve patient compliance.

This study evaluates the usefulness of hand orthosis for the first CMC-OA and compares the 3D-printed orthoses with a conventional orthosis.

## 2. Materials and Methods

### 2.1. Selection Criteria

Among the patients who visited our hospital from 2018 to 2019 and were diagnosed with the first CMC-OA, 12 hands in nine patients (male: 1 hand in one patient, female: 11 hands in eight patients) were included in this study. There were seven cases of dominant hand and five cases of non-dominant hand. Patients with concomitant hand OA other than the first CMC-OA and patients with underlying conditions, such as rheumatoid arthritis, were excluded. The mean age of the patients was 69 years (58–84 years), the mean duration of the disease was 3 years (1–6 years), and the Eaton classification was Stage 2: three hands, Stage 3: eight hands, and Stage 4: one hand. This research has been approved by the IRB of the authors’ affiliated institutions.

### 2.2. Procedure for Orthosis Production

**Figure 1:**
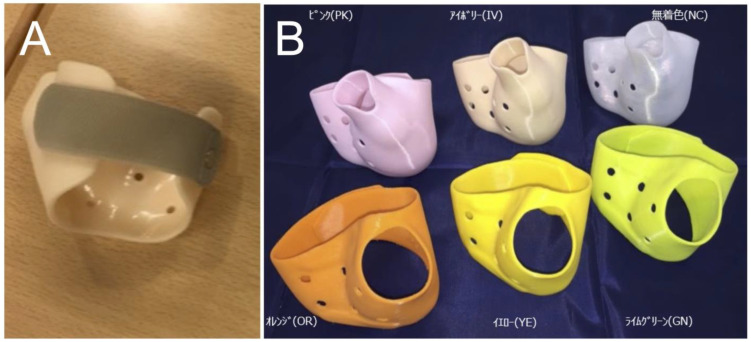
(**A**) Conventional orthosis is made of plastic, with molds taken from each patient by prosthetists. (**B**) Three-dimensional printed orthoses are made of FABREAL^®^, a type of resin and a soft filament developed for 3D-printed orthosis. There were long-type orthoses shown in the upper low and short-type orthoses shown in the lower low.

Prosthetists took a plaster mold from the patient and used the mold to create a rigid conventional plastic orthosis. The area to be fixed was from the palm to the distal part of the metacarpophalangeal joint of the thumb (including the metacarpophalangeal joint and CMC joint) (Figure 1A).

**Figure 2:**
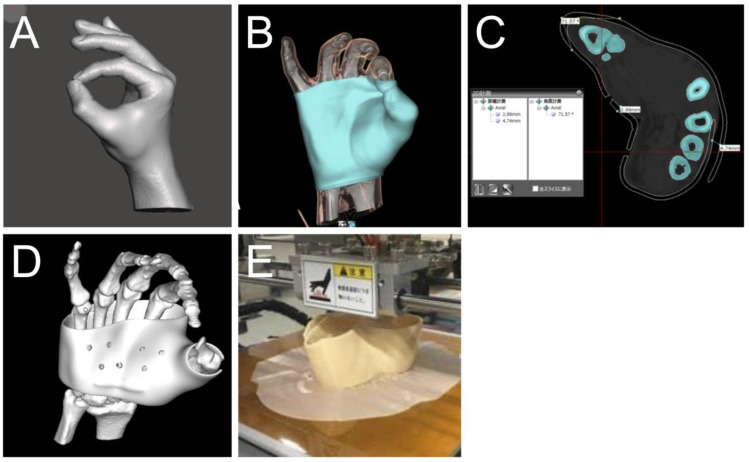
Illustrations show the process of making a 3D-printed orthosis. (**A**) Affected hand in the pinch position was scanned using a 320-row multi-detector CT system. (**B**) The skin surface data and the bone joint surface data are reconstructed. (**C**) Orthosis designed with a thickness of 3 mm in contact with the skin surface. (**D**) Fine-tuning of trim lines and thicknesses is performed while comparing with the bone joint surface data. (**E**) Based on the digital design, each orthosis is formed using Fused Deposition Modeling (3D-printed).

For creating the 3D-printed orthosis (Figure 1B), each affected hand was scanned using a 320-row multi-detector CT system (Aquillion ONE VISION Edition, Toshiba Medical Systems Corporation, Tochigi, Japan). CT scans were performed in a relaxed tip pinch position (Figure 2A). The CT data were accumulated in the Digital Imaging and Communication in Medicine (DICOM) format. Skin surface data and bone joint surface data were reconstructed, and orthoses for each hand were designed using software (Meshmixer version 11, AUTODESK Inc., San Francisco, CA, USA) (Figure 2B–D). The thickness of the orthosis was set at 3 mm, and the area around the joints was slightly thickened to prevent stress concentration. On the dorsal side of the hand, an overlap was made such that it could be fixed with Velcro. Based on the digital design, each orthosis was created using Fused Deposition Modeling (Figure 2E). FABREAL^®^ (JSR Corporation, Tokyo, Japan), a type of resin and a soft filament developed for 3D-printed orthosis was used as the material for the 3D-printed orthosis.

**Figure 3:**
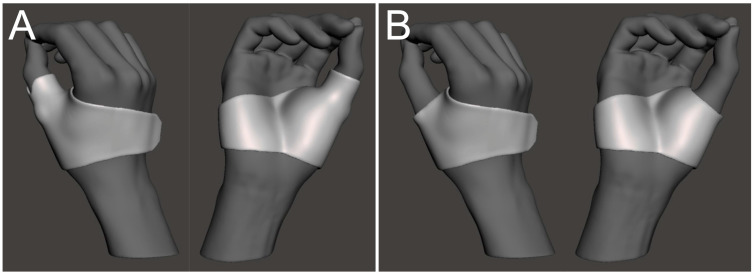
Illustrations show two types of 3D-printed orthosis. (**A**) The long-type includes the MP joint of the thumb, and (**B**) the short-type does not include the MP joint of the thumb.

We designed two different types of fixation area orthoses: a long-type from the palm to the distal of the MP joint of the thumb and a short-type from the palm to the proximal MP joint of the thumb (Figure 1B and Figure 3).

### 2.3. Study Protocol

The participants were instructed to wear three types of orthoses (conventional orthosis, short-type 3D-printed, and long-type 3D-printed orthoses) for 2 weeks each, and changing the type of orthosis worn during the 2-week wearing period was not permitted. The order of wearing the three types of orthoses was randomly assigned based on the case registration number. Participants were encouraged to wear the orthosis for as long as possible during the day and at night. However, they were allowed to wear and take off the orthoses at their discretion. No other additional treatments, such as anti-inflammatory analgesics, injections, or rehabilitative activities, were administered. The participants completed questionnaires before the commencement of the treatment and after wearing each orthosis for two weeks. The questionnaire consisted of the Visual Analogue Scale (VAS) at rest and in motion to assess pain, The Disability of the Arm, Shoulder, and Hand (DASH) questionnaires to assess the function, percentage of time spent using the orthosis during the daytime, patient subjective satisfaction score with each orthosis (0–10 points), and discomfort caused by wearing orthoses (“never”, “occasionally”, or “frequently”). We compared these variables among three types of orthoses.

### 2.4. Statistical Analysis

Pain (VAS at rest and in motion), DASH score (0–100 points), and the time of orthosis usage during the day (0–100%) were treated as continuous variables. Satisfaction score (0–10 points) and discomfort caused by wearing orthoses were treated as ordinal variables. We used one-way repeated analysis of variance (one-way repeated ANOVA) for comparison among orthoses, since the Shapiro–Wilk test results showed normality of distribution only for the DASH score. Furthermore, we used the Friedman test for comparisons of other variables among orthoses. Spearman’s rank correlation coefficient was used to analyze the correlation between satisfaction score and discomfort caused by wearing orthoses. *P*-value less than 0.05 was considered significant for each variable.

## 3. Results

**Figure 4:**
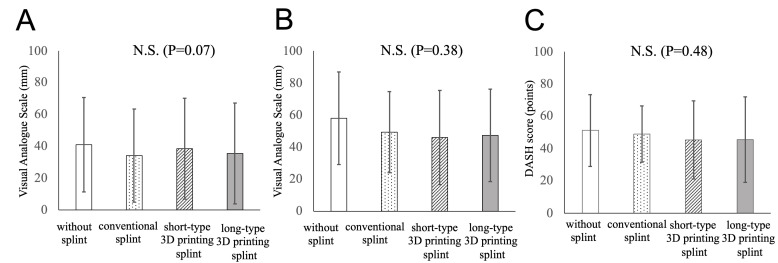
(**A**) Changes in pain scale (visual analog scale: 0–100) before using orthosis and (**B**) after using each orthosis are demonstrated ((**A**): pain at rest, (**B**): pain in motion). The differences among groups are not statistically significant. (**C**) Changes in DASH scores (0–100) before using orthosis and after using each orthosis are determined. The differences among groups are not statistically significant.

All patients completed the protocol. VAS at rest was 41 ± 30 before treatment, 34 ± 29 with a conventional orthosis, 39 ± 32 with a short-type 3D-printed orthosis, and 36 ± 32 with a long-type 3D-printed orthosis, with no statistically significant difference (Figure 4A). VAS in motion was improved from 58 ± 29 to 49 ± 25 before treatment with a conventional orthosis, 46 ± 29 with short-type 3D-printed orthosis, and 47 ± 29 with long-type 3D-printed orthosis; however, the differences were not statistically significant (Figure 4B). The DASH score showed no statistically significant improvement before and after orthosis use (51 ± 22 before treatment, 49 ± 17 with conventional orthosis, 45 ± 24 with short-type 3D-printed orthosis, and 46 ± 26 with long-type 3D-printed orthosis) (Figure 4C).

**Figure 5:**
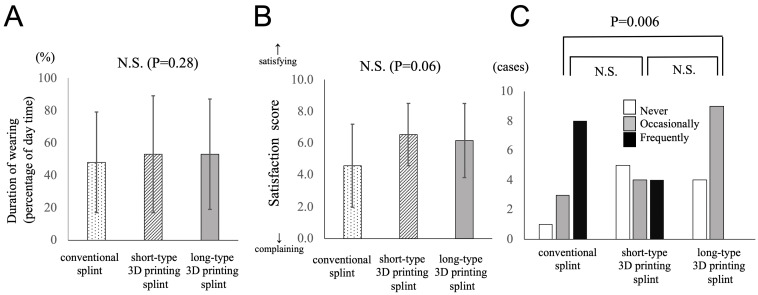
(**A**) Duration of wearing (the percentages of daytime spent) using each orthosis is demonstrated. The differences among the groups are not statistically significant. (**B**) The satisfaction scores (0–10) of each orthosis are shown. The differences among groups are not statistically significant. (**C**) Discomfort frequency (“never”, “occasionally”, or “frequently”) caused by wearing each orthosis is depicted. It was more frequent in the conventional orthosis than in the long-type 3D-printed orthoses significantly. (*p* = 0.006).

The duration of wearing the orthosis during the daytime was 48 ± 31% with conventional orthosis, 53 ± 36% with short-type 3D-printed orthosis, and 53 ± 34% with long-type 3D-printed orthosis. There was no difference among the three types of orthoses (Figure 5A). In the satisfaction score, 3D-printed orthosis was slightly higher than the conventional orthosis when compared on average (4.6 ± 2.6 for the conventional orthosis, 6.5 ± 2.0 for short-type 3D-printed orthosis, and 6.2 ± 2.3 for long-type 3D-printed orthosis), but the differences were not statistically significant (Figure 5B). Discomfort caused by wearing orthosis was more frequent for conventional orthosis than for 3D-printed orthosis. There was a significant difference between the conventional type and the long-type 3D-printed orthosis (*p* = 0.006) (Figure 5C). There was a moderately positive correlation between the satisfaction score and discomfort caused by wearing orthoses (R = 0.51).

## 4. Discussion

In this study, 3D-printed orthosis usage caused less discomfort than conventional orthosis. Because there was a moderately positive correlation between the satisfaction score and discomfort caused by wearing orthoses, relatively high levels of satisfaction were achieved with 3D-printed orthoses, although this was not statistically significant. However, the duration of orthosis usage was approximately half the time during the day for all types of orthoses. This suggests that patient compliance with orthoses was not always good. The high percentage of women who routinely do household chores may have influenced these results in this study. This low wear rate may be a limitation of hand orthosis therapy for the first CMC-OA. However, the duration of orthosis use was assessed solely based on a patient self-reported questionnaire, and a detailed analysis of compliance was not conducted. A more comprehensive evaluation of compliance and usage patterns would require methods such as patient diaries or electronic monitoring.

There were no significant differences in VAS improvement or DASH scores between the three types of orthoses, which shows similar outcomes of 3D-printed orthosis as conventional orthosis. There was a mean reduction of approximately 20% in VAS during motion after 2 weeks of orthosis use, comparable to previous reports. Arazpour M et al. showed a 20% reduction in VAS in 25 patients with the first CMC-OA using a rigid orthosis that did not include the MP joint of the thumb for four weeks [12]. Compared to their study, this study showed comparable pain reduction, although the wearing time in daily life was not standardized and the wearing period was shorter. Because the nighttime wear was not standardized and left to the patient’s decision, it may have contributed to pain reduction. There was no improvement in DASH scores with either orthosis. It is important to note that the DASH score assesses overall upper-limb functionality, which may limit its sensitivity in detecting specific improvements in hand function. This limitation may have contributed to the lack of significant findings in functional improvement. Future studies should consider using hand-specific assessment tools to provide more precise evaluations.

Compared to conventional orthoses, 3D-printed orthoses utilize different materials and different methods for molding and designing. The 3D-printed orthoses were created based on body surface data obtained from CT, which accurately reflected the positional information of the bones and joints for the fixation range. Furthermore, this method easily adjusts the coverage area and thickness of the orthoses. In this study, two orthosis types with different distal trim lines were created and compared. Further verification was performed by adjusting the thickness and fixation area. Regarding the material, the 3D-printed orthoses were made of a FABRIAL, which is a resin-derived filament, whereas the conventional orthoses were made of plastic. FABRIAL is softer than plastic and can be layered using a 3D printer. Reports indicate that the difference in the material of orthoses has a significant effect on the fit and that the fit was better with neoprene than with plastic [13,14]. Differences in materials may contribute to the differences in discomfort caused by wearing the orthosis and satisfaction scores with orthoses.

In this study, two types of 3D-printed orthoses were made: a long-type that includes the MP joint of the thumb and a short-type that does not include the MP joint of the thumb. There was no significant difference between the two types of orthoses in pain relief, functional improvement, satisfaction score, or duration of orthosis wearing. Vanneste M et al. [15] demonstrated that orthoses including the MP joint of the thumb were more effective in immobilizing the first CMC joint adduction and abduction in healthy volunteers. A more distal trim line on the thumb may have a positive effect on the immobilization of the first CMC joint. However, the one that proves more advantageous for patient use is controversial. Cantero-Tellez R et al. [16] reported that the long-type orthoses, including the MP joint of the thumb, offered slightly greater pain relief than the short-type orthoses. However, Buhler M et al. [17] reported no difference in pain and functional improvement between the two types of orthoses. Additionally, some reports concluded that short-type orthoses are often preferable to the long-type [11,18]. Previous studies showed that the production methods for the short and long-types differed, with the short-type being prefabricated and the long-type being custom-made. In this study, the short- and long-type orthoses made using the same methods and materials showed no differences in terms of pain relief, improvement in function, or patient satisfaction. In 3D-printed orthoses, the short-type may be sufficient for the required fixation area but must be examined in a larger number of cases.

This study has several limitations. First, the number of cases was small, and the majority of cases were advanced elderly cases. In comparing the three types of orthoses, the power for the sample size in the current study was 0.64; hence, it is possible that significant differences were not observed because of β error. We have not been able to verify the effectiveness of hand orthosis in adolescent patients because most of the cases in this study were advanced elderly patients. Another limitation is the short follow-up period of only two weeks for each orthosis type. A longer follow-up period is necessary to better assess the sustained effects on pain relief, function, and patient compliance. Future studies should include a larger cohort, stratified by age and severity of CMC-OA, to improve statistical power and generalizability, as well as extended usage periods to capture long-term outcomes. Additionally, three bilaterally affected cases were included in this study. The bilateral condition may have affected orthotic wearing time and satisfaction; however, the small number of cases makes this difficult to analyze. The second limitation involves difficulty in determining whether the results obtained in this study are because of the difference in manufacturing methods or materials. This is because both the manufacturing methods and materials used differ between conventional and 3D-printed orthoses. A study using the same material is required to clarify the effect of 3D-printed orthoses compared to conventional orthoses. Additionally, there was no group with a short conventional orthosis, which would improve the comparison between the groups based on different types of orthoses. Finally, we used a CT image to create the orthosis, which increases medical costs and exposes patients to radiation. CT imaging is approximately 1.5 times more expensive than molding by orthotists. Future research should explore alternative, non-invasive, and cost-effective imaging techniques such as 3D surface scanning, which could help reduce both the financial burden and radiation risks.

## 5. Conclusions

The 3D-printed orthosis made by FABRIAL and digitally designed from CT surface data were used for the first CMC-OA. Although there were no statistically significant differences in clinical outcomes, the comparable effectiveness of 3D-printed orthoses and their reduced discomfort indicate potential applicability in remote healthcare settings. While it remains to be determined whether the benefits justify the additional costs required for computer-designed orthoses, we believe that this technology has potential for future applications in telemedicine and personalized treatment approaches.

## Data Availability

The data presented in this study are available on request from the corresponding author due to ethical reasons.

## References

[1] Swigart C.R., Eaton R.G., Glickel S.Z., Johnson C. (1999). Splinting in the treatment of arthritis of the first carpometacarpal joint. J. Hand Surg. Am..

[2] Kodama R., Muraki S., Oka H., Iidaka T., Teraguchi M., Kagotani R., Asai Y., Yoshida M., Morizaki Y., Tanaka S. (2016). Prevalence of hand osteoarthritis and its relationship to hand pain and grip strength in Japan: The third survey of the ROAD study. Mod. Rheumatol..

[3] Muller G.M. (1949). Arthrodesis of the trapezio-metacarpal joint for osteoarthritis. J. Bone Joint Surg. Br..

[4] Gervis W.H. (1949). Excision of the trapezium for osteoarthritis of the trapezio-metacarpal joint. J. Bone Joint Surg. Br..

[5] Swanson A.B. (1968). Silicone rubber implants for replacement of arthritis or destroyed joints in the hand. Surg. Clin. N. Am..

[6] Wilson J.N. (1973). Basal osteotomy of the first metacarpal in the treatment of arthritis of the carpometacarpal joint of the thumb. Br. J. Surg..

[7] Burton R.I., Pellegrini V.D. (1986). Surgical management of basal joint arthritis of the thumb. Part II. Ligament reconstruction with tendon interposition arthroplasty. J. Hand Surg. Am..

[8] Tsehaie J., Spekreijse K.R., Wouters R.M., Slijper H.P., Feitz R., Hovius S.E.R., Selles R.W. (2018). Outcome of a Hand Orthosis and Hand Therapy for Carpometacarpal Osteoarthritis in Daily Practice: A Prospective Cohort Study. J Hand Surg Am..

[9] Weiss S., LaStayo P., Mills A., Bramlet D. (2000). Prospective analysis of splinting the first carpometacarpal joint: An objective, subjective, and radiographic assessment. J. Hand Ther..

[10] Hamasaki T., Laprise S., Harris P.G., Bureau N.J., Gaudreault N., Ziegler D., Choinière M. (2020). Efficacy of Nonsurgical Interventions for Trapeziometacarpal (Thumb Base) Osteoarthritis: A Systematic Review. Arthritis Care Res..

[11] Grüschke J.S., Reinders-Messelink H.A., van der Vegt A.E., van der Sluis C.K. (2019). User perspectives on orthoses for thumb carpometacarpal osteoarthritis. J. Hand Ther..

[12] Arazpour M., Soflaei M., Ahmadi Bani M., Madani S.P., Sattari M., Biglarian A., Mosallanezhad Z. (2017). The effect of thumb splinting on thenar muscles atrophy, pain, and function in subjects with thumb carpometacarpal joint osteoarthritis. Prosthet. Orthot. Int..

[13] Weiss S., Lastayo P., Mills A., Bramlet D. (2004). Splinting the degenerative basal joint: Custom-made or prefabricated neoprene?. J. Hand Ther..

[14] Sillem H., Backman C.L., Miller W.C., Li L.C. (2011). Comparison of two carpometacarpal stabilizing splints for individuals with thumb osteoarthritis. J. Hand Ther..

[15] Vanneste M., Stockmans F., Vereecke E.E. (2021). The effect of orthoses on the kinematics of the trapeziometacarpal, scaphotrapeziotrapezoidal, and radioscaphoid joints. J. Orthop. Res..

[16] Cantero-Téllez R., Valdes K., Schwartz D.A., Medina-Porqueres I., Arias J.C., Villafañe J.H. (2018). Necessity of Immobilizing the Metacarpophalangeal Joint in Carpometacarpal Osteoarthritis: Short-term Effect. Hand.

[17] Buhler M., Chapple C.M., Stebbings S., Sangelaji B., Baxter G.D. (2019). Effectiveness of splinting for pain and function in people with thumb carpometacarpal osteoarthritis: A systematic review with meta-analysis. Osteoarthr. Cartil..

[18] van der Vegt A.E., Grond R., Grüschke J.S., Boomsma M.F., Emmelot C.H., Dijkstra P.U., van der Sluis C.K. (2017). The effect of two different orthoses on pain, hand function, patient satisfaction and preference in patients with thumb carpometacarpal osteoarthritis: A multicentre, crossover, randomised controlled trial. Bone Joint J..

